# Case of Atrophy Following an Injury of the Right Superior Maxillary Nerve

**Published:** 1847-06

**Authors:** 


*Case of Atrophy following an Injury of the Right Superior Maxillary Nerve.*—The patient, a man aged forty, first consulted M. Vallez, in the middle of August, 1845. In the previous month of May he had felt the power of vision in his right eye decrease progressively. A continual discharge of tears took place from it, changing, after some time, into a puriform discharge. At length, after the application of a poultice, the cornea burst with acute pain. From that time, (about two months before he consulted M. Vallez,) the globe of the eye by degrees became retracted. When M. Vallez first examined the patient, the right eye appeared like an irregular piece of dirty chalk; it performed slight motions, especially when touched or uncovered; it was soft, and induced pain in the bottom of the orbit when pressed. The right side of the face was insensible when pricked with a needle, provided the instrument was not introduced more deeply than the skin. When the patient laughed, his mouth was drawn to the left side, his tongue, also, was displaced towards the left side. His food did not present the same taste to him as before his disease; his smell and hearing were normal. His left eye was unaffected, though rather watery. M. Vallez, while endeavoring to discover the origin of this singular affection, noticed a cicatrix, two inches long, on the right cheek of the patient. This cicatrix followed a line drawn from the right ala of the nose to the lobe of the ear of the same side; it projected, and appeared white and indolent. The cheek of the same side was constantly pale; whilst the left cheek was considerably injected, and possessed its natural degree of heat. The patient stated that, while shaving, a friend had pushed his elbow, causing him to make the wound, which had been very deep, and from which a considerable quantity of blood had flowed. It took a month in healing. The wound took place in the commencement of March, and it was a month after its healing that the disease of the eye commenced.

Our readers are aware that section of the trifacial nerve, as performed by M. Magendie on animals, in the interior of the cranium, always interferes with the nutrition of the eye on the corresponding side, and brings on, after a time, its destruction. Indeed, M. Serres, Dr. Abercrombie, Mr. Stanley, &c., have recorded cases in which the same results have followed the presence of tumors, compressing the trifacial within the cranium, and thus altering its structure in the human subject. In these cases, however, the lesion of the trifacial was situated between the brain and the eye. Cases of amaurosis, following lesions of the peripheral extremity of the nerve, are also well known: such are produced by injury or contusion of the peripheral extremities of branches of the ophthalmic, situated in the temple or the eye-brow; this class of cases forms a distinct order in the description of amaurosis. We have not hitherto, however, on record, any case, as far as we know, in which the nutrition of the globe of the eye has been disturbed in consequence of injury occurring to a part of the nerve placed between the eye and its peripheral extremity.

